# The impact of high knee walking on knee pain and straight leg raising function after patellar fracture surgery: A retrospective study

**DOI:** 10.1097/MD.0000000000047755

**Published:** 2026-03-06

**Authors:** Jiawen Wang, Xiangyu Zhang, Jiahui Li, Xianfeng Zhang, Peng Huang, Jianpeng Zhou, Tianzhao Tian, Yingfeng Cai, Baoxin Liu

**Affiliations:** a Department of Orthopedics, The Affiliated Guangzhou Hospital of TCM of Guangzhou University of Chinese Medicine, Guangzhou, Guangdong Province, China.

**Keywords:** exercise, high knee walking, patellar fracture, postoperative

## Abstract

Open reduction and tension band internal fixation is the standard treatment for patellar fractures. Although this surgery aids early weight-bearing and joint movement, full recovery of knee function – particularly the ability to perform straight leg raising (SLR) – often still requires a longer period, which can affect the patients’ daily activities, such as getting out of bed and dressing. To enhance its recovery, we implemented an early high knee walking (HKW) protocol from June 2023. This retrospective analysis was conducted on 48 patients from June 2023 to April 2025, with 42 eligible patients included in the analysis. Among them, 22 patients initiated HKW training (intervention group) on postoperative day 7, while 20 patients received assisted straight leg raise training (assisted straight leg raising; control group). Self-reported measures assessed at postoperative days 7, 14, 28, and 100 included visual analogue scale score, SLR and seated knee extension (SKE) ability, and thigh circumference difference. The results showed no differences between the 2 groups on postoperative day 7. At 14, 28, and 100 days postoperatively, both groups demonstrated significant improvements compared to baseline (day 7) in all indexes (*P* < .05 or *P* < .01). Additionally, compared to the control group, the intervention group demonstrated quicker pain relief (Day 14, 28, 100; *P* < .05) and swelling reduction (Day 14; *P* < .05), superior SKE (day 28; *P* < .05) and SLR function (day 100; *P* < .05), and less muscle atrophy (day 100; *P* < .01). The results of this study suggest that HKW exercise for the postoperative patients with patellar fractures facilitates pain relief, SLR and SKE recovery, and reduces thigh swelling and muscle atrophy.

## 1. Introduction

Patellar fracture is a typical intra-articular fracture in traumatic orthopedics. Open reduction can make the articular surface close to anatomical recovery, thus reducing the risk of post-traumatic osteoarthritis. Tension band internal fixation reconstructs the continuity of knee extension device, which is convenient for early walking and joint activity, thus helping to prevent joint stiffness and quadriceps muscular atrophy. Therefore, open reduction and tension band internal fixation (ORTBIF) is a recognized operation for patellar fracture at present.^[1-3]^ Clinically, although ORTBIF surgery has largely restored knee flexion and extension activities and walking ability in patients,^[4-6]^ some extending knee movements, such as straight leg raising (SLR) and seated knee extension (SKE), recover slowly, even after a lot of rehabilitative treatment, which causes inconvenience in daily living activities, such as getting up and dressing. Analyzing the reasons, it may be related to knee pain, weakness of the quadriceps and hip flexors after the surgery.^[7-9]^

Early rehabilitation following patellar fracture surgery often involves isometric training of the quadriceps and continuous passive motion with a machine,^[10]^ while SLR exercise can strengthen the quadriceps and activate the hip flexor.^[11]^ From June 2023, we modified the high knee (HK) training including high knee running and standing high knee in sports into high knee walking (HKW) and applied it to the rehabilitation after patellar fracture surgery. Different from the intensity of HK training in physical training, the HKW is a slow movement with the help of a walker. Analyze its movement process, the HKW is a kind of closed dynamic chain movement that drives knee joint to flex and stretch passively through flexion of hip joint.^[12]^ This exercise mode is expected to enhance the muscle strength of quadriceps, activate hip flexion muscles, increase the stability of knee, and enhance the coordination of waist, pelvis and lower limbs.^[13,14]^ This retrospective study aims to evaluate whether the HKW can accelerate pain relief and promote the early recovery of SLR ability and its safety.

## 2. Materials and methods

### 2.1. Study design and population

**Figure 1.**
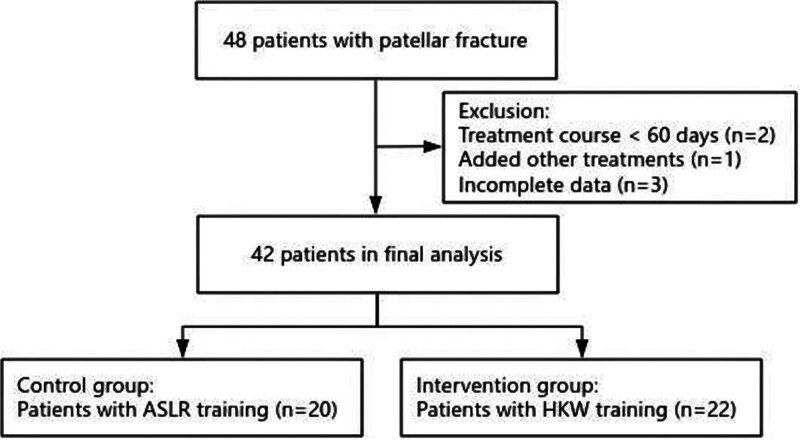
Flowchart of the study participants. HKW = high knee walking, ASLR = assisted straight leg raising.

From June 2023 to April 2025, 48 patients who underwent ORTBIF of patellar fractures in our hospital were screened for qualification. Inclusion criteria: stable and reliable internal fixation suitable for early exercise (evaluated by the surgeon); no visible difference in thickness between the 2 thighs before the injure; on the 7th day postoperatively, vital signs, infection indexes were checked and local incision was stable. On the same day, assisted straight leg raising (control group) or HKW (intervention group) were started. Exclusion: insufficient exercise (<60 days or not enough exercise every day), extra therapy and incomplete data. Finally, 6 patients were excluded (2 patients had insufficient course of treatment, 1 patient joined other treatments, and 3 patients had incomplete data) and 42 patients entered the analysis and were divided into 2 groups according to the treatment methods, 20 cases in the control group and 22 cases in the intervention group. The patient screening process is summarized in Figure 1.

### 2.2. Ethics approval

This retrospective study was approved by the Ethics Review Committee of the Affiliated Traditional Chinese Medicine Hospital of Guangzhou Medical University (No: 2025NK39). Written informed consent for the publication of study data was obtained from all participants.

### 2.3. Interventions

#### 2.3.1. Surgical procedure

All 2-part patellar fracture operations were completed by Kirschner wire, steel wire tension band internal fixation technology and comminuted patella fracture with additional wire hoop ligation, introduced in Campbell’s orthopedic surgery.^[15]^ All the operations were performed by 2 experienced orthopedic surgeons throughout the study period (BX Liu and P. Huang).

#### 2.3.2. Rehabilitation protocol

ASLR: in supine position, one end of the traction belt or elastic belt is fixed on the front foot, and the other end is held in the patient’s hand for traction, which helps the passive/active straight leg raise to the highest point as much as possible. Pause for 10 seconds, then slowly descend to the bed, pause for 3 to 5 seconds, and then start the next cycle. During training, patients should be instructed to exercise autonomously as much as possible, so as to avoid always giving priority to pulling. This exercise includes 2 sets every day, 30 times in each set, and gradually increases to 50 times in each set. If this exercise aggravates the pain, reduce the amount appropriately, and then gradually increase after the pain is relieved.

**Figure 2.**
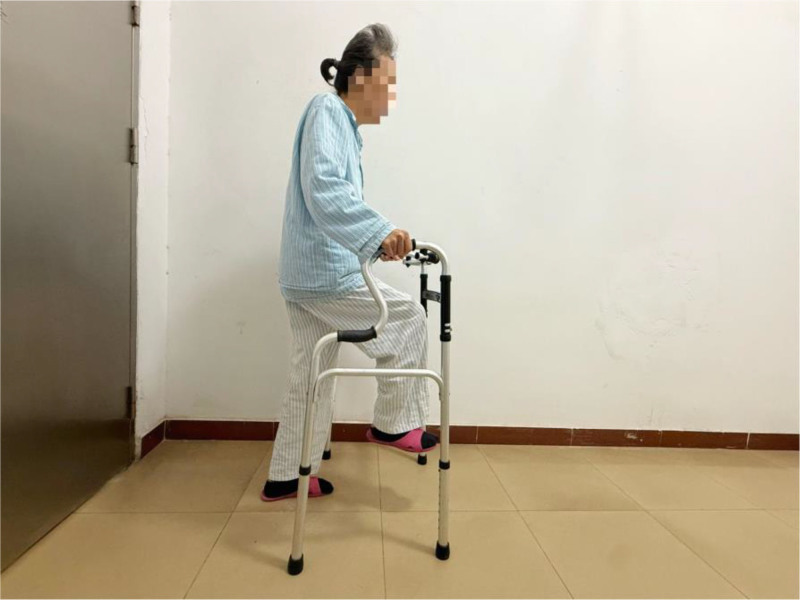
The action of a patient standing with a walker in both hands, keeping her upper body slightly tilted, raising her knees and bending naturally, and stepping forward at the same time under horizontal shooting.

HKW: the patient standing with hands supported by a walker, and the upper body remains upright or slightly inclined. Raise the affected or healthy knee as high as possible, allow the knee to bend automatically, hold at the highest point for 10 seconds, then slowly lower it, step forward at the same time, pause for a moment, and then switch to the other leg to perform the exercise. This exercise is twice a day, with 30 beats per limb, and gradually increases to 50 beats per limb. If this exercise aggravates the pain, then moderate the amount of the exercise, and then gradually increase after the pain is relieved. Figure 2 is a demonstration of the HKW training.

During hospitalization, all the exercises were supervised and guided by trained medical staff. After discharge, patients were regularly assessed via WeChat or outpatient follow-ups to ensure they adhere to the exercise plan punctually and quantitatively.

### 2.4. Self-assessment and training

During hospitalization, trained medical staff provided each patient with a paper form and standardized measurement tools (tape measure and gradiometer with a straight ruler), and offered guidance on correct measurement methods to the patient or their family. The form included basic information and clear self-assessment instructions for each time point, corresponding to the 4 outcome measures.

### 2.5. Outcome measures

The outcome measures included the following: visual analog scale (VAS) score, defined as the pain level marked by patients on a 10cm line (0 for no pain, 10 for the worst pain) and measured in centimeters with a ruler to obtain the pain evaluation value^[16]^; supine SLR ability, evaluated using a binary outcome (yes/no) where a positive result is defined as the patient being able to lift the entire lower limb from the bed to a height of at least 45 degrees of hip flexion while lying flat with the knee fully extended and maintain it for at least 10 seconds; SKE grade, measured by having the patient sit on a high bed or bench with lower legs hanging freely, using a goniometer to determine the maximum active extension angle from the vertical, and classifying performance into excellent (≥75°), good (51–74°), average (26–50°) and poor (≤25°); and thigh circumference difference between the affected and healthy sides, measured with the patient in a supine position at 10 cm proximal to the superior border of the patella, where a larger affected side indicates the affected thigh swelling and a larger healthy side indicates the affected thigh muscle atrophy. All the indicators were assessed on postoperative days 7, 14, 28, and 100 by patients completing self-assessment forms under the guidance of trained medical staff.

### 2.6. Data collection

General data were extracted from electronic medical records, including demographic information, fracture characteristics, surgical details, and length of hospital stay. Self-reported outcome data were collected from the forms completed by the patients on postoperative days 7, 14, 28, and 100.

### 2.7. Statistical analysis

Data were analyzed using SPSS 25.0. (Cary) continuous variables are presented as mean ± standard deviation. Normality and homogeneity of variance were tested. Independent samples *t* tests and chi-squared test were used for baseline characteristics between 2 groups. VAS scores were compared both within and between groups using repeated measures ANOVA with Bonferroni post hoc tests. Chi-squared test was used to analyze the number of patients able to perform active SLR, Mann–Whitney *U* test was used to analyze SKE grades, and independent sample *t* tests was used to analyze thick circuit differences for 2 groups. A significance level of *P* < .05 was adopted for all tests.

## 3. Results

### 3.1. Comparisons of general clinical data between 2 groups

Comparisons of baseline characteristics between the 2 groups are presented in Table 1. No statistically differences were observed in age, gender, fracture type, cause of injury, time from injury to surgery, operation time, blood loss, or length of hospital stay (*P* > .05). However, a significant difference was noted in the distribution of the affected side (L/R) between 2 groups (*P* = .003). Despite this imbalance, the groups were considered broadly comparable for the purpose of this retrospective analysis.

**Table 1:** General data of participating patients.

Characteristic	Control group (N = 20)	Intervention group (N = 22)	*P*-value
Age (yr), mean ± SD	56.55 ± 15.49	61.18 ± 16.24	.351*
Gender (M/F), n	9/ 11	8/ 14	.569†
Side (L/R), n	7/ 13	18/ 4	.002†
Fracture type (two-part/comminuted), n	7/ 13	6/ 16	.589†
Cause of injury (traffic accident/fall), n	8/ 12	6/ 16	.382†
Time from injury to surgery (<24 h/>24 h), n	19/ 1	19/ 3	.341†
Operation time (min), mean ± SD	60.20 ± 7.01	60.18 ± 6.88	.993*
Blood loss (ml), mean ± SD	62.25 ± 26.13	60.68 ± 17.88	.820*
Length of hospital stay (d), mean ± SD	15.75 ± 9.00	11.64 ± 7.66	.117*
Treatment course (d), mean ± SD	60–93 (81.65 ± 10.62)	61–92 (79.77 ± 9.63)	.553*

SD = standard deviation.

* *t*-test.

† Chi-squared test.

### 3.2. Clinical outcomes

#### 3.2.1. Complications

No complications such as worsened pain or wound iridescence were observed in either group.

#### 3.2.2. Comparisons of the VAS scores for 2 groups

On postoperative day 7, there was no significant difference in VAS scores between the 2 groups (*P* = .160). However, the intervention group demonstrated significantly lower VAS scores compared to the control group at postoperative day 14 (*P* = .012), day 28 (*P *= .029), and day 100 (*P* = .047; Table 2).

**Table 2:** Pre- and post-intervention comparison of VAS scores by group.

Group	Day 7	Day 14	Day 28	Day 100	*F*	*P*
Control group (N = 20)	4.19 ± 0.26	3.45 ± 0.21	2.82 ± 0.44	1.19 ± 0.52	194.742	.000
Intervention group (N = 22)	4.30 ± 0.26	3.20 ± 0.37	2.53 ± 0.40	0.86 ± 0.51	377.081	.000
*t*	1.430	2.636	2.262	2.046		
*P*-value	.160	.012	.029	.047		
Group*Test Times					3.048	.039

Bonferroni post hoc tests. Control group: versus Day 7, all *P *< .01; Intervention group: versus. Day 7, all *P *< .01.

VAS = visual analogue scale.

#### 3.2.3. Comparisons of the number of patients able to perform active SLR for 2 groups

No significant differences were observed between the intervention and control groups on postoperative day 7 (*P* = .493), day 14 (*P* = .524), or day 28 (*P* = .187). However, at day 100, a significantly higher proportion of patients in the intervention group were able to perform active SLR compared to the control group (*P* = .027; Table 3).

**Table 3:** Comparison of the number of patients able to perform active SLR by group.

Group	Day 7	Day 14	Day 28	Day 100
Control group (N = 20), n (%)	2 (10.00)	3 (15.00)	6 (30.00)	14 (70.00)
Intervention group (N = 22), n (%)	1 (4.55)	5 (22.73)	11 (50.00)	21 (95.45)
χ^2^	0.470	0.406	1.739	4.887
*P*-value	.493	.524	.187	.027

SLR = straight leg raising.

#### 3.2.4. Comparisons of SKE grades for 2 groups

No significant differences were found between the intervention and control groups at postoperative day 7 (*P* = .464) or day 14 (*P* = .862). A statistically significant difference in favor of the intervention group was observed at day 28 (*P *= .016). By day 100, the difference between groups was no longer statistically significant (*P* = .127; Table 4).

**Table 4:** Comparison of SKE grades by group.

Time point	Control group (N = 20) (excellent/good/fair/poor)	Intervention group (N = 22) (excellent/good/fair/poor)	*Z*	*P*-value
Day 7	0/9/11/0	0/10/11/1	0.733	.464
Day 14	0/12/8/0	3/15/4/0	0.174	.862
Day 28	2/14/4/0	7/15/0/0	2.417	.016
Day 100	16/4/0/0	21/1/0/0	1.526	.127

SKE = seated knee extension.

#### 3.2.5. Comparisons of TC differences for 2 groups

Prior to postoperative day 28, the affected thigh was larger in both groups, indicating swelling. No significant between-group difference was observed at postoperative day 7 (*P* = .216). At postoperative day 14, the intervention group showed a significantly smaller difference (less swelling) compared to the control group (*P* = .015). By postoperative day 28, swelling had largely resolved in both groups, with no significant between-group difference (*P* = .656). At postoperative day 100, the affected thigh was smaller in both groups, indicating muscle atrophy, which was significantly more pronounced in the control group (*P* < .001; Table 5).

**Table 5:** Comparison of the TC differences by group.

Group	Day 7	Day 14	Day 28	Day 100
Control group (N = 20), mm	−6.90 ± 1.21	−4.40 ± 1.14	0 ± 0.09	7.10 ± 1.17
Intervention group (N = 22), mm	−6.27 ± 1.91s	−3.32 ± 1.55	−0.14 ± 0.99	5.05 ± 1.09
*t*	−1.258	−2.547	0.449	5.904
*P*-value	.216	.015	.656	.000

TC = thigh circumference.

## 4. Discussion

ORTBIF is a common surgical procedure for patellar fractures, providing a foundation for early rehabilitation. However, achieving complete recovery of knee function postoperatively remains challenging.^[17]^ Among various functions, supine SLR ability is particularly difficult to restore. This study evaluated the efficacy of HKW in reducing knee pain and restoring supine SLR ability, while also examining its effects on SKE and reduction of thigh muscles atrophy. The results demonstrated that at 14-, 28- and 100-days post-operation, the intervention group showed superior outcomes compared to the control group, with significantly reducing knee pain VAS scores, improving supine SLR ability, enhancing SKE, and reducing thigh swelling and thigh muscles atrophy.

High knee exercise is an auxiliary training method used in many sports, which can be divided into high knee running and in situ high knee training. This exercise uses body weight to activate multiple muscle groups, mainly the quadriceps, hip flexors, glutes, hamstrings, and calves. It not only strengthens muscles but also improves body coordination and balance.^[18]^ High knee training is a kind of high-intensity physical training, and the body needs good balance ability, otherwise it is easy to fall. Clinically, we improved it into HKW, that is, walking slowly with the help of a walker, which greatly improved the safety of this training. This study applied HKW to the rehabilitation after patellar fracture surgery and found that no exercise-related complications occurred. In addition, compared with bed training, patients are more willing to exercise on the ground, so HKW improves patients’ compliance.

SLR ability is a fundamental lower limb ability in humans, requiring coordinated efforts of the quadriceps (especially the rectus femoris), iliopsoas, and abdominal muscles. During this movement, hip flexors (primarily the iliopsoas) provide the lifting force, while the quadriceps maintain knee extension through isometric contraction, with the patella serving as a mechanical pivot.^[19-21]^ Although SLR ability does not affect walking on flat ground, it does impact daily activities such as getting up, dressing, and climbing stairs, etc. After patellar fracture surgery, despite reconstruction of the extensor mechanism, restoring SLR ability remains challenging. Among the 42 patients in this study, only 3 could perform SLR on the postoperative day 7, and only 8 cases were able to perform this movement by postoperative day 14. By postoperative day 100, 7 patients still had not regained this ability. A similar phenomenon has been observed after total knee arthroplasty.^[22]^ Initially, we attributed this to knee swelling and pain.^[23]^ However, even after the resolution of swelling and pain, some patients still could not perform active SLR, leading us to consider muscle incoordination in the lower limbs as a contributing factor, necessitating coordinated rehabilitation training.^[24–26]^

HKW combines high-intensity active muscle movement with passive knee flexion and extension. Compared to the control group, it led to significantly faster pain relieving and swelling reducing from postoperative day 14 onward (*P* < .05), although no significant difference was observed on day 7 (*P* = .160), creating a prerequisite for the recovery of SLR ability. Concurrently, the high-intensity lower limb exercise effectively prevented adhesion between muscle fibers. At postoperative day 100, thigh muscle atrophy was observed in both groups but was significantly more pronounced in the control group (*P* < .001). The high-intensity lower limb exercise regimen reduced muscle atrophy, prevented adhesion between muscle fibers, improved knee stability, and provided dynamic support for restoring straight leg raise (SLR) ability. Most importantly, HKW involves complex coordination among multiple muscle groups in the lumbar-abdominal region and lower limbs.^[27,28]^ Long-term practice of this exercise benefits the recovery of SLR ability and promotes the restoration of SKE, as demonstrated in this study.

Nevertheless, several limitations of this study should be acknowledged. First, this is a retrospective study, and the imbalance in the left/right injured knee ratio between the 2 groups could not be fully controlled (*P* = .003); second, patient-reported outcomes are highly subjective and may be biased; finally, the relatively small sample size may limit the generalizability of the findings and reduce the statistical power to detect smaller effect sizes. Future research requires larger sample sizes and more rigorously controlled randomized controlled trials to further confirm the findings.

## 5. Conclusions

HKW exercise can accelerate pain relief and swelling reduction, improve the recovery of SLR and SKE abilities, and reduce thigh muscle atrophy in patients after patellar fracture surgery.

## Acknowledgments

We would like to thank all the patients who provided information in this study.

## Author contributions

**Data curation:** Jiawen Wang, Jiahui Li, Peng Huang.

**Formal analysis:** Jianpeng Zhou, Tianzhao Tian, Yingfeng Cai.

**Methodology:** Peng Huang.

**Supervision:** Baoxin Liu.

**Validation:** Xianfeng Zhang.

**Writing – original draft:** Jiawen Wang, Xiangyu Zhang.

**Writing – review & editing:** Baoxin Liu.
